# Avenanthramides, Distinctive Hydroxycinnamoyl Conjugates of Oat, *Avena sativa* L.: An Update on the Biosynthesis, Chemistry, and Bioactivities

**DOI:** 10.3390/plants12061388

**Published:** 2023-03-21

**Authors:** Chanel J. Pretorius, Ian A. Dubery

**Affiliations:** Research Centre for Plant Metabolomics, Department of Biochemistry, University of Johannesburg, Auckland Park 2006, South Africa

**Keywords:** alkaloid, avenanthramide, biomarkers, conjugates, hydroxyanthranilic acid, hydroxycinnamic acid, metabolomics, *N*-hydroxycinnamoyltransferase

## Abstract

Avenanthramides are a group of *N*-cinnamoylanthranilic acids (phenolic alkaloid compounds) that are produced in oat plants as phytoalexins, in response to pathogen attack and elicitation. The enzyme catalysing the cinnamamide-generating reaction is hydroxycinnamoyl-CoA: hydroxyanthranilate *N*-hydroxycinnamoyltransferase (HHT, a member of the super family of BAHD acyltransferases). HHT from oat appears to have a narrow range of substrate usage, with preferred use of 5-hydroxyanthranilic acid (and to a lesser extent, other hydroxylated and methoxylated derivatives) as acceptor molecules, but is able to use both substituted cinnamoyl-CoA and avenalumoyl-CoA thioesters as donor molecules. Avenanthramides thus combine carbon skeletons from both the stress-inducible shikimic acid and phenylpropanoid pathways. These features contribute to the chemical characteristics of avenanthramides as multifunctional plant defence compounds, as antimicrobial agents and anti-oxidants. Although avenanthramides are naturally and uniquely synthesised in oat plants, these molecules also exhibit medicinal and pharmaceutical uses important for human health, prompting research into utilisation of biotechnology to enhance agriculture and value-added production.

## 1. Introduction

A broad range of natural products are synthesised by plants, often through specialised and species-specific metabolic pathways. These pathways are supported by primary/core metabolism, that provides the precursors, scaffolding molecules, and co-factors that enable and support specialised metabolite biosynthesis; together, forming metabolic networks involved in the synthesis of structurally complex and functionally diverse molecules [1]. Functions of these diverse groups of phytochemicals are often associated with plant communication, protection, and adaptation to environmental signals. Remarkably, the biosynthesis of the many phenolic, terpene, fatty acid-derived, and nitrogen-containing phytochemicals is dependent on only a few major pathways, with the large diversity of these metabolites being achieved by elaboration of the core carbon skeletons, using second tier enzymes that catalyse oxidation/reduction, decarboxylation, hydroxylation, methylation, glycosylation, and acylation reactions [2,3]. One of the most common types of modification of secondary metabolites such as phenolics, is the acylation of substrates containing oxygen- and/or nitrogen, to generate esters and amides, respectively [4]. Acylation affects different properties of these metabolites, and can influence the function of phenolic compounds *in planta* [4,5].

Oat (*Avena sativa* L.) is an eco-friendly grain crop, with numerous nutritional, medicinal and pharmaceutical uses. Numerous phytochemicals with high antioxidant, anti-inflammatory, and antiproliferative properties are produced by wild, as well as cultivated, oat plants. These include flavonoids, phenolics, saponins, tocopherols, and tocotrienols. Among the cultivated species, white oat (*A. sativa*) and red oat (*A. byzantina*) are the only grain crops that synthesise avenanthramides (Avns) [6,7,8]. Avns have thus been regarded as signature compounds in oat. Here, the biosynthesis and structural and functional diversity of these unique oat phytochemicals, are further elaborated on, in the sections that follow.

## 2. Avenanthramides as Distinctive Oat Phytochemicals

Avenanthramides are a group of phenolic alkaloid compounds that are produced in oat plants, as the hydroxyanthranilic acid (hydroxylated 2-amino-benzoic acids) derivatives or conjugates of hydroxycinnamic acids. Oat bran and flakes contain the largest concentration of Avns that are generally present in all milling fractions, and thus found in commercial oat products [9,10,11]. To date, at least forty different Avns (both glycosylated and non-glycosylated derivatives) have been found and identified in oat plants and products, with Avns A, B, and C being the most abundant in differentiated tissues [12,13]. Avn synthesis exhibits tissue-specific peculiarities, with constitutive expression in grain (endosperm and scutellum) and inducible expression in leaves [14]. Moreover, biosynthesis is activated at different developmental stages [15]. For example, the authors of [16] reported a 25 fold increase in the Avn content from seed to seedling, and with a different Avn profile to that found in extracts from oat seeds. Interestingly, undifferentiated cell suspension cultures of oat apical meristem callus tissue were demonstrated to synthesise Avns A and G in response to chitin elicitation [17]. Under certain culture conditions, Avn B and C were also produced, as well as three additional metabolites, tentatively identified as Avn H, O, and R (nomenclature explained in Section 4). The differences in the Avn compositions of the leaf tissue, compared to that of the callus tissue, may be related to the differentiation state of the cells.

## 3. Biosynthesis of Avenanthramides

One of the most frequent types of modification of secondary metabolites, is the acylation of substrates containing oxygen- and/or nitrogen, to generate esters and amides, respectively [4,5]. In such reactions, the activated acyl donors are derived from sources such as acyl-sugars, acylated acyl carrier proteins, or acyl-activated coenzyme A thioesters [5].

The BAHD acyltransferases are CoA-dependent enzymes, that transfer acylated moieties (RC(O)R’) of an acyl-activated CoA thioester donor to an acceptor molecule [2]. Members of this BAHD enzyme family characteristically share two conserved amino acid domains. In the first motif, the sequence of consecutive amino acids is HXXXD(G) (in the central region of the enzyme), with the histidine acting as a catalytic residue. The second conserved motif is DFGWG, typically found near the C-terminus of the protein and thought to be involved in CoA binding [2]. Interestingly, the DFGWG motif is located distal to the active site of the enzyme and apparently does not participate in the catalytic mechanism. It was suggested that the oxygen or nitrogen atom on the corresponding acceptor substrate is deprotonated by the histidine, permitting a nucleophilic attack on the carbonyl carbon of the CoA thioester. This, in turn, forms a tetrahedral intermediate between the CoA thioester and the acceptor substrate. The intermediate subsequently becomes re-protonated, to produce the acylated ester or amide, together with the free CoA [2].

Phylogenetic analyses using full-length amino acid sequences of functionally characterised BAHD enzymes, indicate five distinctive clades that correlate to general function [2,18]. BAHD acyl transferases generally exhibit low substrate selectivity, and can use a variety of CoA thioester and alcohol co-substrates (e.g., aromatic and aliphatic alcohols) and amines, glycosides, terpenoids, and alkaloids [2]. The acronym BAHD refers to the first letter of the first four enzymes that were discovered to belong to this family, with ‘H’ representing the *N*-hydroxycinnamoyl/benzoyltransferase (HCBT) found in clade Vb [18]. HCBT was the first identified case of a BAHD member that is capable of transferring the acyl moiety to a nitrogen atom to form the corresponding amide, and was identified in elicited cell cultures of carnation, *Dianthus caryophyllus* [19].

The catalytic versatility of BAHD enzymes makes it challenging to make functional predictions from primary sequence alone. Some BAHD members have a restricted range of substrate usage, whereas others have wide substrate specificity in vitro, such that the products they form *in planta* are sometimes determined by the relative availability of substrates. Relatedly, motif enrichment analysis and molecular dynamics simulations of BAHD acyltransferase family members, suggest that such specialisation may originate from amino acid sequence variations concentrated in specific motifs, rather than from variations that are globally distributed throughout the enzyme structure [20,21]. In the case of the biosynthesis of Avns from oat, the major enzyme in this process is hydroxycinnamoyl-CoA: hydroxyanthranilate *N*-hydroxycinnamoyltransferase (HHT, E.C. 2.3.1.302, a member of the super family of BAHD acyltransferases), with the first *HHT* genes (1–4) cloned by [22].

The process of Avn biosynthesis begins with the production of *trans*-cinnamic acids from phenylalanine, by phenylalanine ammonia-lyase (PAL), and the hydroxylation of the cinnamate to p-coumaric acid, in the presence of cinnamic acid 4-hydroxylase (C4′H). Following its conversion into its activated CoA thioester analog, by 4-coumarate-CoA ligase (4CL), the resulting p-coumaroyl-CoA (donor molecule) is then conjugated to 5-hydroxyanthranilic acid (acceptor molecule), catalysed by HHT, to generate Avn A. This is followed by a number of reactions catalysed by various enzymes. Relatedly, p-coumaroyl-CoA is frequently transformed to p-coumaroyl shikimate or quinate, before being hydroxylated by p-coumaroyl CoA ester 3′-hydroxylase to generate caffeoyl-CoA.

**Figure 1:**
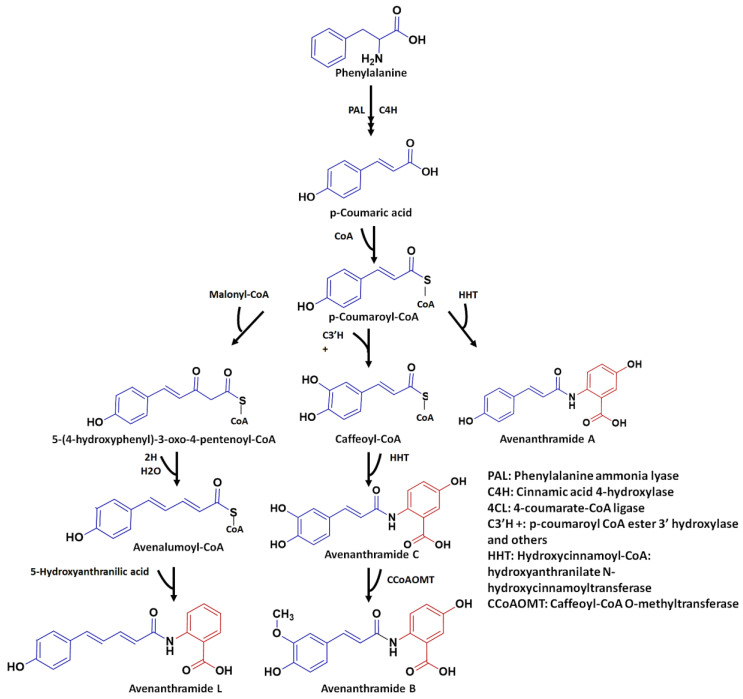
Schematic of the proposed biosynthetic pathway of the three major avenanthramides A, B, and C (hydroxycinnamic acid derivatives) and avenanthramide L (an avenalumin derivative) in oat, that are composed of an anthranilic acid (AA, red) and a phenylalkenoic acid (PA, blue). The PA moiety originates from the stress-inducible phenylalanine ammonia lyase in the early phenylpropanoid pathway, while the anthranilic acid originates from the shikimate pathway via chorismate. The conjugating enzyme is a hydroxycinnamoyl CoA: hydroxyanthranilate *N*-hydroxycinnamoyltransferase (HHT), that catalyses the *N*-acylation of hydroxyanthranilates with various activated hydroxycinnamoyl-CoAs as aroyl-moieties, to form a series of Avns. Avns are thus *N*-cinnamoylanthranilic acids; with p-coumaroyl-CoA yielding Avn A and caffeoyl-CoA yielding Avn C. Avn B is derived from Avn C through methylation. The alternative use of avenalumic acid, through avenalumoyl-CoA as donor molecule, generates Avn L.

The biosynthesis of these compounds (Avn A, B, and C) was previously believed to be catalysed by a single HHT enzyme, able to accept all the substituted cinnamoyl-CoA thioesters [14,22,23]. However, new results [24] have demonstrated that two HHT enzymes catalyse the *N*-acylation of 5-hydroxyanthranilic acid with p-coumaroyl-CoA or caffeoyl-CoA, but not with feruloyl-CoA, indicating that oat HHTs are only involved in the biosynthesis of Avn A (as a coumaroyl derivative) and Avn C (a caffeoyl derivative), but not Avn B. In this scenario the enzyme caffeoyl-CoA *O*-methyltransferase (CCoAOMT), then methylates Avn C to produce Avn B (the ferulic acid derivative) [24] (Figure 1).

Similarly, the proposed biosynthesis of avenalumic acid, starts off with the condensation of p-coumaroyl-CoA and malonyl-CoA, to form 5-(4-hydroxyphenyl)-3-oxo-4-pentenoyl-CoA. This intermediate is then reduced and dehydrated to avenalumoyl-CoA, which is then converted to Avn L in the presence of 5-hydroxyanthranilic acid [25]. The avenalumic acids can be regarded as ethylenic homologues of p-coumaric, caffeic, and ferulic acids [26].

Regulation of the biosynthesis of specialised plant metabolites, might involve chromosome decondensation, histone and DNA modification, as well as the formation of biosynthetic gene clusters and metabolons [1]. Incomplete and inconclusive information on the biosynthesis of Avns has hindered genetic improvement of this important nutritional trait in oat [24]. Cultivated oat (*A. sativa* L.) is an allohexaploid, with three subgenomes and a basic chromosome number of x = 7 (AACCDD, 2n = 6x = 42). The recent sequencing and annotation of the *A. sativa* genome, will support the development of varieties that meet the increasing global demand for oat-derived products [27]. As an allohexaploid plant, it may mainly contains duplicated genes [28], and the sequences of six HHTs genes (*AsHHT 1–6*) were reported as part of an investigation of the biosynthetic pathway of the major Avns [24]. All six gene sequences exhibited the two conserved motifs, identical at an amino acid level. However, due to a lack of genome-wide characterisation, insights into gene transcription, regulation, and expression profiling, it is not known if each copy of these duplicated genes is uniformly expressed in different tissues and under different conditions [28]. To add to the layered complexity, differential substrate specificities of HHT isoforms might affect the type and concentration of Avns found in different tissue types of oat.

## 4. Diversity of Avenanthramide Structures and Associated Nomenclature

Hydroxycinnamates are among the most widely distributed plant phenylpropanoids, present as free, conjugated-soluble and insoluble-bound forms. As conjugated-soluble forms, the common moiety of the structures of Avn metabolites are composed of the phenylalkylenoic acid (PA), connected with an amide bond to an anthranilic acid (AA) (the aminobenzoic acid, 5-hydroxyanthranilic acid) [29]. To a lesser extent, the AA moiety may also be supplied as 5-hydroxyanthranilic acid, 5-hydroxy-4-methoxyanthranilic acid, 4-hydroxyanthranilic acid, or 4,5-dihydroxyanthranilic acid [30]. As such, Avns may occur as blends of closely related members, that differ in the number and position of hydroxyl and methoxy substituent groups.

**Figure 2:**
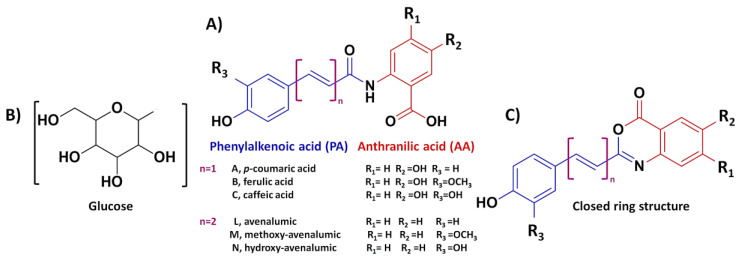
General structure and Collins nomenclature of avenanthramides, illustrating the phenylalkenoic acid (PA) moiety in blue and the anthranilic acid (AA) moiety in red (**A**). Glycosylation of Avns can also occur through the addition of glucose to free hydroxyl groups (**B**), which expands the range of reported Avns. (**C**) Closed benzoxazinone ring structure of an ‘avenalumin’.

The chemical diversity of the Avn structures is further expanded due to the fact that the Avns (AA–PA) can contain a PA component, that is either a hydroxycinnamic acid or an avenalumic acid [6,14,26]. Compared to the hydroxycinnamic acid derivatives (C_6_–C_3_), the lesser known avenalumic acid ((2*E*,4*E*)-5-(4-hydroxyphenyl)-penta-2,4-dienoic acid) contains an extended side chain (C_6_–C_5_), with an additional double bond (diene homologues), and can also have extra hydroxy and methoxy substituents [26,31], similar to hydroxycinnamic acids (Figure 2). In addition, Avn aglycones can occur as glycosides, when glucose attaches to free hydroxyl groups on either the AA or PA module [30], this can greatly expand the chemical diversity of Avns present in oat.

Collins and colleagues were among the first to report and name the diverse groups of Avns, using an alphabetical naming system [12,32]. Later, the systematic nomenclature system was modified, to denote the AA subunit (e.g., A = anthranilate, B = 5-hydroxy anthranilate), and a letter to denote the PA subunit; c (caffeic acid), f (ferulic acid), or p (p-coumaric acid) [33]. Longer-chained PA subunits (avenalumins) were denoted by adding an extra subscripted letter (example: 2f_d_) [34]. Table 1 provides a list of natural Avns, based on their nomenclature as constructed by Collins and Dimberg [32,33], with their respective R groups as illustrated in Figure 2.

Historically, the phytoalexins found by [31], were named as avenalumins I, II, and III, which were later recognised as the closed ring intramolecular esters (4H-3,1-benzoxazin-4-ones) (Figure 2C) of the hydrated open ring amides corresponding to p-coumaric -, ferulic -, and hydroxyl-avenalumic acids, respectively. The term avenalumin is no longer in use and has been replaced with avenanthramide [14].

Both the total content (range of 0.55–775 mg/kg fresh weight) and the levels of individual Avns, vary greatly between cultivars and oat products [30].

## 5. Avenanthramide Extraction and Detection

It is unfortunate that, due to bio-analytical challenges, the chemical profile and concentrations of Avns are not well reported. Over 40 distinct Avns have been detected, in anionic fractions from 80% aqueous methanol extracts of oat grain [12], of which the structures of 10 could be elucidated. Given the modular composition of Avns, it is possible that further combinations of the AA and PA moieties, with other substitution patterns and sugar linkages may exist, generating novel Avn structures. The number of known Avns is increasing, due to technological developments in bio-analytical techniques. In this regard, 29 Avns (aglycones and glucosides), extracted with 50% ethanol, were recently identified and characterised by liquid chromatography (LC), mass spectrometry (MS), and nuclear magnetic resonance (NMR) [30]. Of these, 17 novel Avn glucosides were reported for the first time. Using UHPLC, with ion trap (IT) MS and high resolution electro-spray (ESI)-MS, 28 unique Avns, in extracts from seedlings, were reported [16]. Relatedly, using high-resolution tandem mass spectrometry (MS/MS), 35 Avns could be identified and characterised in oat seed extracts, and the structures of 10 novel Avns elucidated [35].

AVA extractions have been mostly water–alcohol based, for their solubilisation with common extractants, including 80% methanol, 80% ethanol, and 50% ethanol. Due to differential solubilities, the type of extractant used can have an effect on the concentration and number of avenanthramides extracted. Seventy percent methanol, was found to extract high concentrations of avenanthramides A, B, and C [36]. In a study by [37], different extraction ratios were tested, to determine the effect on the concentration of extracted avenanthramides (A, B, and C). The best extraction conditions were different for each of the three primary Avns. Moreover, the ideal alcohol composition varied between Avns. Avn A showed better extraction with high alcohol concentrations (86–100% *v*/*v*), whereas Avn C required the lowest alcohol concentration (54% *v*/*v*). Collectively, the optimum extractant concentrations for the Avns range from 50–100%, largely affected by the different hydroxycinnamic acid moieties present. Depending on the starting material, some protocols have been adjusted to include an additional defatting step, prior to alcohol extraction, using hexane or petroleum ether to remove neutral and polar lipids, respectively [38,39,40].

## 6. Post-Synthesis Alterations: Isomerisation and Dimerisation

As explained above, hydroxycinnamic acids (p-coumaric, caffeic, and ferulic acids) are important constituents that make up the respective Avn structures. Cinnamic acids have been known to undergo *trans* (*E*) to *cis* (*Z*) isomerisation, when exposed to UV radiation, due to the presence of a conjugated styryl function in the C_6_–C_3_ skeleton [12,41]. Therefore, when exposed to UV light or sunshine, Avns can undergo similar photo-isomerisation, changing from the naturally formed *E* isomer to the *Z* isomer, and generating isomeric pairs of the respective Avns [12,26]. Avns with longer-chained PA derivatives (i.e., Avns with avenalumic acid moieties) undergo *E*-Z isomerisation of the two double bonds in the PA subunit. These longer-chained Avns can therefore theoretically exist in four different isomers: *E-E*, *E-Z*, *Z-E*, and *Z-Z*. However, the most abundant of the isomers is the naturally occurring *E-E* isomer [16,34]. Knowledge of the stereoisomeric configuration of bound phenolic acids is frequently a necessary prerequisite to evaluating their functional properties. Moreover, geometric isomerisation results in differences in retention times during reverse phase chromatography, and together with the differences in substitution patterns, adds to the complexity of profiling Avns present in oat extracts [12].

Another structural change that has been reported, is the formation of bisAvn B from Avn B through dimerisation. The dimerisation of compounds resulting in oligomers, that are often present together with their monomers, is not a new concept in plants. Dehydrodimers of ferulic acids have been reported in oat, barley, and rye [9]. Serotonin dimers in barley [42], and Avn B dimers in oats, are likely produced by comparable radical coupling processes. The radical coupling dimerisation reaction can produce a novel skeleton, that is entirely distinct from the precursor; as a result, it significantly adds to the diversity of secondary metabolites from oat [43]. In some of these cases, the dimerisation of a compound can result in stronger antimicrobial activity than that of the original monomer. Furthermore, these dimeric substances can often have a role distinct from the original monomer. An example of such of a distinctly different function is cell-wall strengthening, where ether-bond dimers between two ferulic acids or feruloyltyramine bridges are found when linked to cell wall structures [4]. As such, a chemical deterrent in response to pathogen attack can also support the process of strengthening physical barriers against pathogen ingress, through cross-linking.

**Figure 3:**
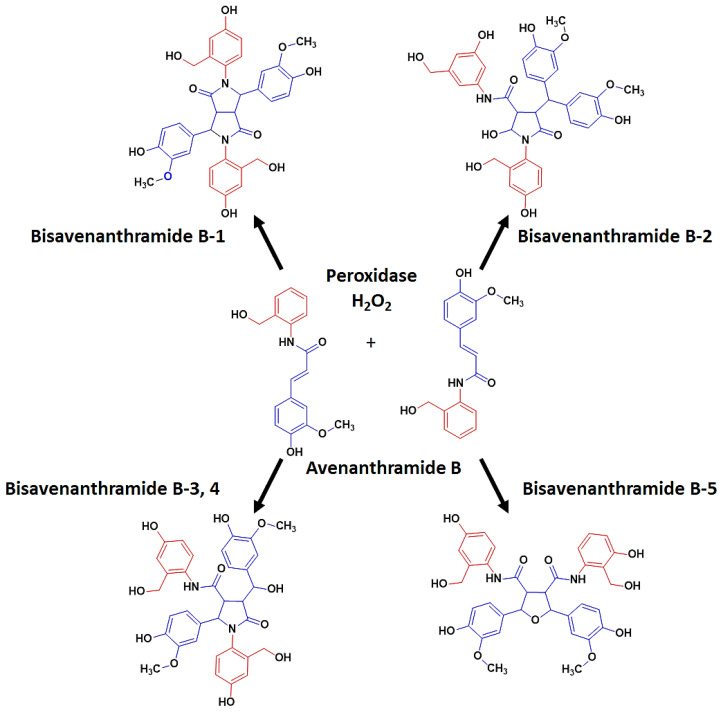
Representation of the proposed synthesis of bisavenanthramides **B1**–**B5** from avenanthramide B. Avn B units are coupled in the presence of peroxidase and hydrogen peroxide, and triggered by the production of phenoxy radicals. **B1** is a dehydrodimer of Avn B, with a bisbutane lactam skeleton, while 2–4 are monohydrated dehydrodimers, with butane lactam structures (**B2**–**B4**), or a tetrahydrofuran structure (**B5**) (adapted from [44]). Ferulic acid and anthranilic acid moieties are indicated in blue and red, respectively.

In oat crops, five dehydrodimers of Avn B have been identified [44], known as bisAvn B. These Avn dimers have been named bisAvn B1 to B5 (all ferulic acid dehydrodimers containing two amide groups) and classified as lignanamides. It was described that in most cases, peroxidases catalyse the 8′-8′ coupling reaction between two Avn B units, in radical coupling processes. Peroxidases do occur in the oat apoplast [45], and hydrogen peroxide is generated as part of the oxidative burst in the apoplast upon pathogen infection. The bisAvns are depicted in Figure 3. These are the only Avn dimers that have been detected to date, and no other Avns (other than B) have been found to result in the formation of dimers [44].

## 7. *In Planta* Bioactivities of Avenanthramides

Functions of these Avns (both hydroxycinnamic acid and avenalumic acid variants) are often associated with plant protection, and adaptation to environmental stressors, to protect plants from infection, disease, and herbivore attack, by acting as antimicrobial agents or chemical barriers [46]. Initial research on the biosynthesis of Avns, focused on their production in vegetative tissue in response to fungal elicitation (especially *Puccinia coronata* f. sp. *avenae* that causes crown rust in oat), but it was later found to be induced by chemical elicitors, chemical mimics of pathogen infection, and certain abiotic stressors [14,47]. Little research, however, has been performed on the production of Avns during bacterial and viral infections. Several bacteria are known to infect oat crops, with the most common being *Pseudomonas syringae* pv. *coronafaciens* (halo blight), *Pseudomonas syringae* pv. *striafaciens* (bacterial stripe blight), and *Xanthomonas compestris* pv. *translucens* (black chaff/leaf streak). The most common viruses to infect oat crops include oat blue dwarf marafivirus (oat blue dwarf), oat mosaic bymovirus (oat mosaic), and oat necrotic mottle tritimovirus (oat necrotic mottle) [48].

Phytoalexins (produced in reaction to pathogen attack) and phytoanticipins (produced before pathogen infection) are two types of phytochemical deterrents/antimicrobial agents commonly produced in plants, when exposed to pathogens [49]. Avns are commonly found as both phytoanticipins and phytoalexins in oat seedlings and plants [50,51]. In this context as phytoprotectants, it is of interest that the Avn profile of seedlings differs from that of seeds, where the Avns A (2p), C (2c), and B (2f), frequently described as the major Avns in oat grains, were shown to represent less than 20% of the total Avn content in the seedlings [16]. Avns were initially identified as phytoalexins in oat leaves infected by pathogenic fungus *Puccina coronata* f. sp. Ave [31], and it has since been established that Avns serve as phytoalexins, with antibacterial or antifungal properties [10,31,44].

Recently, an untargeted metabolomics study (based on ultra-high performance LC coupled to high-definition quadrupole MS) of oat seedlings against the halo blight disease bacterial pathogen, *Pseudomonas syringae* pv. *coronafaciens,* revealed the induction of Avns as discriminatory biomarkers, linked to the host defence response [51]. In general, the protective action of phytoalexins is attributed to a combinatory effect of their cellular localisation during the host response to the pathogen attack, their timeous and sustained bioavailability, the upregulation of specific metabolites over others among the same subfamily, and/or putative synergistic effects between these metabolites. Accordingly, although not fully understood, their mode of action on microorganisms arises from an interference with important cellular structures and metabolism [52]. Additionally, upon infection, they can be covalently integrated into the cell wall to provide protection against breakdown, which frequently happens as a result of the enzymes released by pathogens [29].

Limited studies have been published on the biosynthesis of Avns in oat, regarding the elucidation of the underlying mechanisms that are involved in the synthesis of these phytoalexins as part of the host defence response [14,24,25]. In support of the antimicrobial activity, Avns also exhibit potent anti-oxidant and radical scavenging activities that, in addition to the cinnamic acid structure, also involves the hydroxyanthranilic acid moiety [29,53]. Structure–activity relationship results revealed that the substitution pattern on both aromatic rings of the AA and PA moieties plays a role, and that both catechol and guaiacyl substitution, as well as carboxyl groups, are involved [54].

## 8. Health-Beneficial Properties of Avenanthramides

In the case of humans, Avns are known to be important as a component of oat as a functional food, where they function as phytonutrients/nutraceuticals in concert with dietary fibres, to provide protective and health-beneficial effects against the emergence of a number of diseases. These include cardiovascular diseases [55], diabetes [56], inflammatory bowel disease [57], cancer [58], obesity [59], and celiac disease [60]. In addition, the Avns exhibit dermatological effects, such as anti-inflammatory and anti-itch activity [61]. The therapeutic benefits and potential of natural and synthetic Avns were reviewed in [10].

Although Avns are naturally and uniquely synthesised in oat plants as defence compounds [62], this type of translational research can act as a foundation for the synthesis of more focused and complex compounds, such as Tranilast, *N*-(3′,4′-di-methoxycinnamoyl)-anthranilic acid. Tranilast is a synthetic anthranilate, where one of the anilino hydrogens is replaced by a 3,4-dimethoxycinnamoyl group. It is a member of cinnamamides, a dimethoxybenzene, an amidobenzoic acid, and a secondary carboxamide. This pharmaceutical was first synthesised by Kissei Pharmaceuticals and approved for use in Japan and Korea as a treatment for inflammatory diseases such as allergic conjunctivitis, bronchial asthma, keloids, dermatitis, and hypertrophic scars [63]. In addition, the drug has also been observed to be beneficial against a variety of diseases such as fibrosis, cancer, cardiovascular diseases, ocular and renal diseases, diabetes, and proliferative disorders. A closely related natural compound, *N*-(4′-hydroxy-3′-methoxycinnamoyl) anthranilic acid (Avn E), was reported as a lipoxygenase inhibitor [12].

Regarding Avns as *N*-cinnamoylanthranilic acids, it is notable that the presence of functional head groups on anthranilic acid and some analogues (starting material for the synthesis of heterocyclic compounds and integrated into many plant alkaloids), enable their conjugation and derivatisation for developing rationally designed molecules, and for studying anthranilic acid-based libraries, for identifying potential pharmacophores. At present, the anthranilates and some structural analogues are important components in numerous bioactive compounds and commercialised drugs, and a wide range of associated biological activities show promise in the management of several diseases and in regulating the disease causing pathways (reviewed by [64]).

## 9. Biotechnological Approaches for Avenanthramide Synthesis

These beneficial health-promoting/nutraceutically important properties, have largely contributed to research into the biosynthesis of these compounds in microbial organisms for biotechnological purposes. The synthesis of high-value secondary metabolites in production hosts (e.g., microbial cells or cultured plant cells), offers many opportunities and has generated significant interest. Metabolic manipulation, or “rational metabolic-flow switching strategies” [65], may allow for the production of exogenous metabolites, such as the Avns, in heterologous hosts. One such approach [66] was followed, using *Escherichia coli* and a tyrosine overproducing strategy. In this study, they managed to produce Avn D and Avn F, that can be used as a framework to improve Avn production, using combinatorial methods that are environmentally friendly. In a study by [23], the genes that are required for the production of Avns were incorporated into *E. coli*, in order to produce nine different Avns. They also altered *E. coli* to produce more substrates for the production of Avns, and managed to produce Avn F, E, and D from glucose, and Avn A and G, by adding hydroxyanthranilate. This study paved the way to investigating the diverse Avns and their biological activities in greater detail.

## 10. Concluding Remarks and Future Perspectives

As phenolic alkaloids, Avns are important specialised secondary metabolites in oat, especially as multifunctional plant protective phytochemicals. For this reason, these compounds have been investigated, to elucidate their role in plant defence. Moreover, research into the health beneficial properties associated with human consumption of oat and oat-based products is becoming increasingly relevant. Nonetheless, to date, the scope of studies of Avns have been limited and not vastly expanded, leaving many gaps and opportunities for future research to explore. As the basis of future investigations, there is a need for improved analytical techniques for the profiling, identification, and quantification of the Avns.

In addition to antimicrobial activities, their role(s) as anti-oxidants, involved in host defence and responses to abiotic environmental stressors, should be further investigated, as part of a systems biology approach, with a focus on genotype–environment–phenotype (G × E × P) interactions, demonstrating the interrelationship among the various components. Here, detailed genomic and integrated multi-omic data can contribute to insights regarding the developmental and environmental regulation of Avn metabolism in oat. Research on the production of these phytochemicals under various conditions deserves further exploration, since past studies have been focussed mainly on the production of these compounds under fungal elicitation, chemical elicitation, and some abiotic stresses. Research on the biosynthesis of Avns in response to pathogens other than fungi, for instance, has not been explored in the past. This type of research could provide deeper insights and expand on the functions of these phytochemicals in plant defence, and subsequently be used to improve crop resistance. Moreover, the biosynthesis of Avns presents remarkable features, where carbon skeletons derived from glycolysis and the pentose phosphate pathway combine in the shikimic acid pathway, to generate hydroxycinnamic acids and hydroxyanthranilic acids as co-substrates for the HHT conjugation reaction, to generate the alkaloid compounds. Little is known about the inducibility of the HHT enzyme(s) and regulation of the carbon flux through precursor pathways in support of Avn biosynthesis. Such knowledge will contribute to the manipulation and improvement of the pathways involved, through modern breeding and biotechnological approaches.

From the viewpoint of Avns as plant-derived bioactives, further studies are necessary to fully address the reported beneficial effects of Avns on human health. These include the characterisation of molecules and mechanisms that mediate the pleiotropic effects of Avns in cellular and animal models of human diseases. Specifically, useful insights could be derived from omics approaches, guided by the identification of predictive bio-markers.

## Figures and Tables

**Table 1:** The nomenclature of oat avenanthramides, with n and R groups corresponding to the respective avenanthramide structures, as indicated in Figure 2.

Collins [32] Nomenclature	Dimberg [33] Nomenclature	n	R_1_	R_2_	R_3_
A	2p	1	H	OH	H
B	2f	1	H	OH	OCH_3_
C *	2c	1	H	OH	OH
L	1p_d_	2	H	H	H
D	1p	1	H	H	H
F	1c	1	H	H	OH
E	1f	1	H	H	OCH_3_
N	1c_d_	2	H	H	OH
M	1f_d_	2	H	H	OCH_3_
O	2p_d_	2	H	OH	H
Q	2c_d_	2	H	OH	OH
P	2f_d_	2	H	OH	OCH_3_
X	3p	1	OCH_3_	OH	H
Z	3c	1	OCH_3_	OH	OH
Y	3f	1	OCH_3_	OH	OCH_3_
U	3p_d_	2	OCH_3_	OH	H
W	3c_d_	2	OCH_3_	OH	OH
V	3f_d_	2	OCH_3_	OH	OCH_3_
G	4p	1	OH	H	H
K	4c	1	OH	H	OH
H	4f	1	OH	H	OCH_3_
R	4p_d_	2	OH	H	H
T	4c_d_	2	OH	H	OH
S	4f_d_	2	OH	H	OCH_3_
AA	5p	1	OH	OH	H
BB	5f	1	OH	OH	OCH_3_
CC	5c	1	OH	OH	OH
OO	5p_d_	2	OH	OH	H
QQ	5c_d_	2	OH	OH	OH
PP	5f_d_	2	OH	OH	OCH_3_

* Thus Collins’ Avn C = Dimberg’s Bc or 2c. The most common Avns are esters of 5-hydroxyanthranilic acid with p-coumaric acid (2p/A), caffeic acid (2c/C), or ferulic acid (2f/B).

## Data Availability

No new data were created or analysed in this study. Data sharing is not applicable to this article.
